# Linking dental pathologies and cancer *via* Wnt signalling

**DOI:** 10.18632/oncotarget.22281

**Published:** 2017-11-03

**Authors:** Pierfrancesco Pagella, Claudio Cantù, Thimios A. Mitsiadis

**Affiliations:** Thimios A. Mitsiadis: Orofacial Development & Regeneration, Institute of Oral Biology, University of Zurich, Zurich, Switzerland

**Keywords:** Wnt signalling, Bcl9, enamel, tooth, cancer

## In search of ideal therapeutic targets

Great efforts have been made over the past decades to discover new therapeutic targets for a big variety of human pathologies. Most of the studies dealing with severe pathological conditions such as cancers and tissue malformations are focused on the role of either widely recognized master controlling genes such as *ras* and *myc* or pivotal components of key signalling pathways, among which Wnt and Notch [1, 2]. However, these genes and molecules are fundamental for proper embryogenesis as well as for tissue and organ homeostasis and regeneration, where they regulate cell proliferation, migration, differentiation and apoptosis. The precise timing and localization of their activation are important to ensure the appropriate cellular functions in physiological conditions. Thus, their indiscriminate targeting is not desirable, due to both the high risk of severe side effects and the certainty of broad phenotypic consequences. On the contrary, optimal therapeutic targets should be selected based on their tissue, time and pathology specific roles. Here, we suggest a paradigmatic example of such target molecules that could be represented by the Wnt/β-catenin signalling components Bcl9 and Bcl9l.

## New candidates in dental pathologies

**Figure 1:**
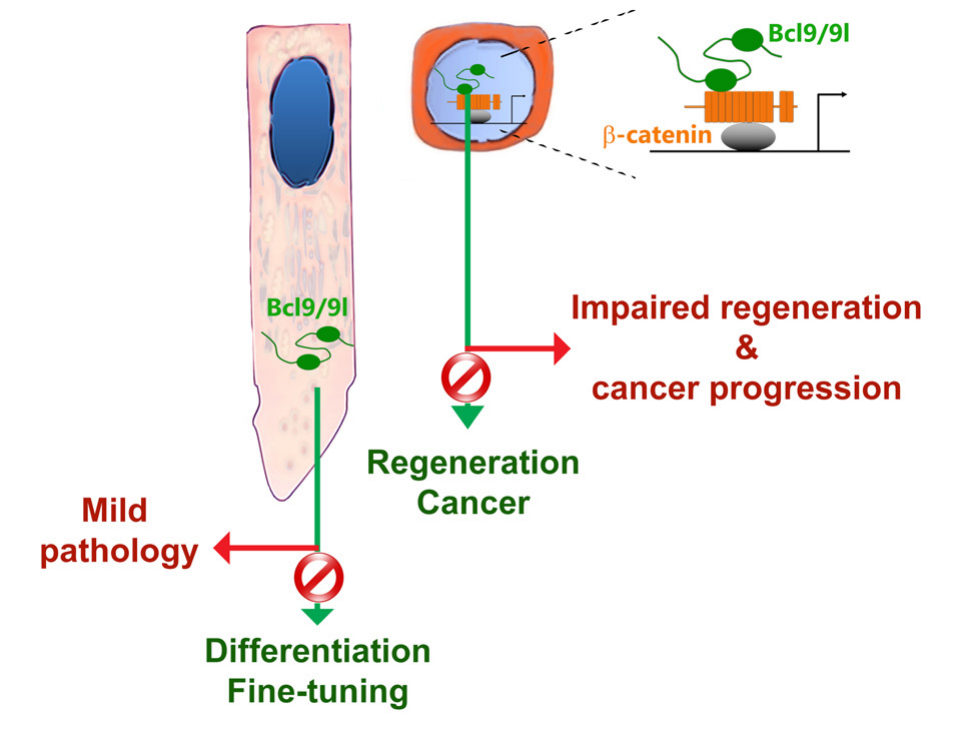
Schematic model summarizing the roles of Bcl9/9l in development and disease according to their nuclear or cytoplasmic localization.

Our recent studies performed in rodent teeth showed that the transcriptional modulators of the Wnt signalling pathway, Bcl9 and Bcl9l (Bcl9/9l), possess a role in the fine-tuning of the tooth enamel [3]. The peculiar structural and chemical organization of the enamel, which is the most mineralized and hardest tissue of the human body, is of crucial importance for its ability to resist insults such as mechanical wear, stress, bacterial toxins and acids that can lead to various dental pathologies. Despite their clinical relevance, the molecular mechanisms responsible for dental defects and the observed variability in tooth properties in humans are essentially unknown [4]. Mutations in *Bcl9/9l* in the mouse affect the fine structure of enamel, thus making it more fragile and likely prone to bacterial infiltrations that facilitate caries progression [3]. In ameloblasts, which are the cells responsible for enamel matrix formation, Bcl9/9l possess a cytoplasmic function, quite in contrast to their previously recognized role as transcriptional activators (Figure 1). In dental tissues, together with their known binding partner Pygo, they interact with secretory vesicles to modulate the secretion and maturation of the enamel matrix. This newly discovered function of Bcl9/9l strongly suggests that genetic variations might influence the predisposition to dental defects related to enamel quality in humans. The cytoplasmic pathway driven by Bcl9/9l appears to be a specialized tissue-specific mechanism that regulates the fine-tuning of enamel but not the overall formation of teeth. We suggest that this mechanism might represent an ideal target for diagnostic and possibly therapeutic interventions aiming at preserving the structural stability of enamel [3].

## From teeth to cancer

Bcl9/9l are also widely expressed in other tissues, such as the intestinal epithelium. Here, they fine-tune the expression of Wnt targets, including genes involved in stem cell function [5]. Similarly to the effects observed in teeth, conditional deletion of *Bcl9/9l* in the intestinal epithelium does not exert dramatic effects on its development and homeostasis [5, 6]. However, in injured intestinal epithelium, the absence of Bcl9/9l impairs its regeneration that causes higher mouse mortality [6]. This effect is of extreme importance as it is now well established that the same mechanisms responsible for tissue regeneration are abnormally activated in several cancers [7]. Cancers could be seen as wounds that never heal and therefore the molecular mechanisms involved in tissue regeneration may play a leading role in tumour progression and metastasis [7]. Consistently, the loss of Bcl9/9l in colorectal cancers has striking effects on their progression and metastatization [6, 8].

Taken together these findings indicate that Bcl9/9l modulate and fine-tune tissue-specific processes. Their activation and inhibition lead to consequences that do not affect homeostatic conditions but impair/debilitate cell-specific responses required in tissue fine-tuning during regeneration and pathology. Bcl9/9l represent promising targets as their action fall precisely within the so-called “therapeutic window”. Challenging protein networks important in a regenerative context could constitute an innovative experimental approach aimed at identifying relevant targets hijacked during tissue-specific diseases and cancers. The ideal molecular targets should reside within the set of molecules whose perturbations do not affect homeostasis, but modulate tissue regeneration and cancer. Their particular mechanism and specificity make Bcl9/9l a paradigmatic example of this view. Our findings might indicate that many other proteins, commonly considered as mere tissue-specific effectors, could exert specific and variable functions that would make them optimal therapeutic targets for tissue-specific pathologies.
